# “Homework Should Be…but We Do Not Live in an Ideal World”: Mathematics Teachers’ Perspectives on Quality Homework and on Homework Assigned in Elementary and Middle Schools

**DOI:** 10.3389/fpsyg.2019.00224

**Published:** 2019-02-19

**Authors:** Pedro Rosário, Jennifer Cunha, Tânia Nunes, Ana Rita Nunes, Tânia Moreira, José Carlos Núñez

**Affiliations:** ^1^Departamento de Psicologia Aplicada, Escola de Psicologia, Universidade do Minho, Braga, Portugal; ^2^Departamento de Psicología, Universidad de Oviedo, Oviedo, Spain

**Keywords:** perceived quality homework, homework characteristics, math, teachers’ perspectives, elementary school, middle school, focus group, homework samples

## Abstract

Existing literature has analyzed homework characteristics associated with academic results. Researchers and educators defend the need to provide quality homework, but there is still much to be learned about the characteristics of quality homework (e.g., purposes, type). Acknowledging that teachers play an important role in designing and assigning homework, this study explored teachers’ perspectives regarding: (i) the characteristics of quality homework and (ii) the characteristics of the homework tasks assigned. In the current study, mathematics teachers from elementary and middle schools (*N* = 78) participated in focus group discussions. To enhance the trustworthiness of the findings, homework tasks assigned by 25% of the participants were analyzed for triangulation of data purposes. Data were analyzed using thematic analysis for elementary and middle school separately. Teachers discussed the various characteristics of quality homework (e.g., short assignments, adjusted to the availability of students) and shared the characteristics of the homework tasks typically assigned, highlighting a few differences (e.g., degree of individualization of homework, purposes) between these two topics. Globally, data on the homework tasks assigned were consistent with teachers’ reports about the characteristics of the homework tasks they usually assigned. Findings provide valuable insights for research and practice aimed to promote the quality of homework and consequently students’ learning and progress.

## Introduction

The extensive literature on homework suggests the importance of completing homework tasks to foster students’ academic achievement (e.g., Trautwein and Lüdtke, 2009; Hagger et al., 2015; Núñez et al., 2015a; Valle et al., 2016; Fernández-Alonso et al., 2017). However, existing research also indicate that the amount of homework assigned is not always related to high academic achievement (Epstein and Van Voorhis, 2001; Epstein and Van Voorhis, 2012). In the words of Dettmers et al. (2010) “homework works if quality is high” (p. 467). However, further research is needed to answer the question “What is quality homework?”.

Teachers are responsible for designing and assigning homework, thus our knowledge on their perspectives about this topic and the characteristics of the homework typically assigned is expected to be a relevant contribution to the literature on the quality of homework. Moreover, data on the characteristics of homework could provide valuable information to unveil the complex network of relationships between homework and academic achievement (e.g., Cooper, 2001; Trautwein and Köller, 2003; Trautwein et al., 2009a; Xu, 2010).

Thus, focusing on the perspective of mathematics teachers from elementary and middle school, the aims of the present study are twofold: to explore the characteristics of quality homework, and to identify the characteristics of the homework tasks typically assigned at these school levels. Findings may help deepen our understanding of why homework may impact differently the mathematics achievement of elementary and middle school students (see Fan et al., 2017).

### Research Background on Homework Characteristics

Homework is a complex educational process involving a diverse set of variables that each may influence students’ academic outcomes (e.g., Corno, 2000; Trautwein and Köller, 2003; Cooper et al., 2006; Epstein and Van Voorhis, 2012). Cooper (1989, 2001) presented a model outlining the factors that may potentially influence the effect of homework at the three stages of the homework process (i.e., design of the homework assignment, completion of homework and homework follow-up practices). At the first stage teachers are expected to consider class characteristics (e.g., students’ prior knowledge, grade level, number of students per class), and also variables that may influence the impact of homework on students’ outcomes, such as homework assignment characteristics. In 1989, Cooper (see also Cooper et al., 2006) presented a list of the characteristics of homework assignments as follows: amount (comprising homework frequency and length), purpose, skill area targeted, degree of individualization, student degree of choice, completion deadlines, and social context. Based on existing literature, Trautwein et al. (2006b) proposed a distinct organization for the assignment characteristics. The proposal included: homework frequency (i.e., how often homework assignments are prescribed to students), quality, control, and adaptivity. “Homework frequency” and “adaptivity” are similar to “amount” and “degree of individualization” in Cooper’s model, respectively. Both homework models provide a relevant theoretical framework for the present study.

Prior research has analyzed the relationship between homework variables, students’ behaviors and academic achievement, and found different results depending on the variables examined (see Trautwein et al., 2009b; Fan et al., 2017). For example, while homework frequency consistently and positively predicted students’ academic achievement (e.g., Trautwein et al., 2002; Trautwein, 2007; Fernández-Alonso et al., 2015), findings regarding the amount of homework assigned (usually assessed by the time spent on homework) have shown mixed results (e.g., Trautwein, 2007; Dettmers et al., 2009; Núñez et al., 2015a). Data indicated a positive association between the amount of homework and students’ academic achievement in high school (e.g., OECD, 2014a); however, this relationship is almost null in elementary school (e.g., Cooper et al., 2006; Rosário et al., 2009). Finally, other studies reported a negative association between time spent on homework and students’ academic achievement at different school levels (e.g., Trautwein et al., 2009b; Rosário et al., 2011; Núñez et al., 2015a).

Homework purposes are among the factors that may influence the effect of homework on students’ homework behaviors and academic achievement (Cooper, 2001; Trautwein et al., 2009a; Epstein and Van Voorhis, 2012; Rosário et al., 2015). In his model Cooper (1989, 2001) reported instructional purposes (i.e., practicing or reviewing, preparation, integration and extension) and non-instructional purposes (i.e., parent-child communication, fulfilling directives, punishment, and community relations). Depending on their nature, homework instructional purposes may vary throughout schooling (Muhlenbruck et al., 2000; Epstein and Van Voorhis, 2001). For example, in elementary school, teachers are likely to use homework as an opportunity to review the content taught in class, while in secondary school (6th–12th grade), teachers are prone to use homework to prepare students for the content to be learned in subsequent classes (Muhlenbruck et al., 2000). Still, studies have recently shown that practicing the content learned is the homework purpose most frequently used throughout schooling (e.g., Xu and Yuan, 2003; Danielson et al., 2011; Kaur, 2011; Bang, 2012; Kukliansky et al., 2014). Studies using quantitative methodologies have analyzed the role played by homework purposes in students’ effort and achievement (Trautwein et al., 2009a; Rosário et al., 2015, 2018), and reported distinct results depending on the subject analyzed. For example, Foyle et al. (1990) found that homework assignments with the purposes of practice and preparation improved the performance of 5th-grade students’ social studies when compared with the no-homework group. However, no statistical difference was found between the two types of homework purposes analyzed (i.e., practice and preparation). When examining the homework purposes reported by 8th-grade teachers of French as a Second Language (e.g., drilling and practicing, motivating, linking school and home), Trautwein et al. (2009a) found that students in classes assigned tasks with high emphasis on motivation displayed more effort and achieved higher outcomes than their peers. On the contrary, students in classes assigned tasks with high drill and practice reported less homework effort and achievement (Trautwein et al., 2009a). A recent study by Rosário et al. (2015) analyzed the relationship between homework assignments with various types of purposes (i.e., practice, preparation and extension) and 6th-grade mathematics achievement. These authors reported that homework with the purpose of “extension” impacted positively on students’ academic achievement while the other two homework purposes did not.

Cooper (1989, 2001) identified the “degree of individualization” as a characteristic of homework focused on the need to design homework addressing different levels of performance. For example, some students need to be assigned practice exercises with a low level of difficulty to help them reach school goals, while others need to be assigned exercises with high levels of complexity to foster their motivation for homework (Trautwein et al., 2002). When there is a disparity between the level of difficulty of homework assignments and students’ skills level, students may have to spend long hours doing homework, and they may experience negative emotions or even avoid doing homework (Corno, 2000). On the contrary, when homework assignments meet students’ learning needs (e.g., Bang, 2012; Kukliansky et al., 2014), both students’ homework effort and academic achievement increase (e.g., Trautwein et al., 2006a; Zakharov et al., 2014). Teachers may also decide on the time given to students to complete their homework (Cooper, 1989; Cooper et al., 2006). For example, homework may be assigned to be delivered in the following class (e.g., Kaur et al., 2004) or within a week (e.g., Kaur, 2011). However, research on the beneficial effects of each practice is still limited.

Trautwein et al. (2006b) investigated homework characteristics other than those previously reported. Their line of research analyzed students’ perception of homework quality and homework control (e.g., Trautwein et al., 2006b; Dettmers et al., 2010). Findings on homework quality (e.g., level of difficulty of the mathematics exercises, Trautwein et al., 2002; homework “cognitively activating” and “well prepared”, Trautwein et al., 2006b, p. 448; homework selection and level of challenge, Dettmers et al., 2010; Rosário et al., 2018) varied regarding the various measures and levels of analysis considered. For example, focusing on mathematics, Trautwein et al. (2002) concluded that “demanding” exercises improved 7th-grade students’ achievement at student and class levels, while “repetitive exercises” impacted negatively on students’ achievement. Dettmers et al. (2010) found that homework assignments perceived by students as “well-prepared and interesting” (p. 471) positively predicted 9th- and 10th-grade students’ homework motivation (expectancy and value beliefs) and behavior (effort and time) at student and class level, and mathematics achievement at class level only. These authors also reported that “cognitively challenging” homework (p. 471), as perceived by students, negatively predicted students’ expectancy beliefs at both levels, and students’ homework effort at student level (Dettmers et al., 2010). Moreover, this study showed that “challenging homework” significantly and positively impacted on students’ mathematics achievement at class level (Dettmers et al., 2010). At elementary school, homework quality (assessed through homework selection) predicted positively 6th-grade students’ homework effort, homework performance, and mathematics achievement (Rosário et al., 2018).

Finally, Trautwein and colleagues investigated the variable “homework control” perceived by middle school students and found mixed results. The works by Trautwein and Lüdtke (2007, 2009) found that “homework control” predicted positively students’ homework effort in mathematics, but other studies (e.g., Trautwein et al., 2002, 2006b) did not predict homework effort and mathematics achievement.

### The Present Study

A vast body of research indicates that homework enhances students’ academic achievement [see the meta-analysis conducted by Fan et al. (2017)], however, maladaptive homework behaviors of students (e.g., procrastination, lack of interest in homework, failure to complete homework) may affect homework benefits (Bembenutty, 2011a; Hong et al., 2011; Rosário et al., 2019). These behaviors may be related to the characteristics of the homework assigned (e.g., large amount of homework, disconnect between the type and level of difficulty of homework assignments and students’ needs and abilities, see Margolis and McCabe, 2004; Trautwein, 2007).

Homework is only valuable to students’ learning when its quality is perceived by students (Dettmers et al., 2010). Nevertheless, little is known about the meaning of homework quality for teachers who are responsible for assigning homework. What do teachers understand to be quality homework? To our knowledge, the previous studies exploring teachers’ perspectives on their homework practices did not relate data with quality homework (e.g., Xu and Yuan, 2003; Danielson et al., 2011; Kaur, 2011; Bang, 2012; Kukliansky et al., 2014). For example, Kukliansky et al. (2014) found a disconnect between middle school science teachers’ perspectives about their homework practices and their actual homework practices observed in class. However, results were not further explained.

The current study aims to explore teachers’ perspectives on the characteristics of quality homework, and on the characteristics underlying the homework tasks assigned. Findings are expected to shed some light on the role of teachers in the homework process and contribute to maximize the benefits of homework. Our results may be useful for either homework research (e.g., by informing new quantitative studies grounded on data from teachers’ perspectives) or educational practice (e.g., by identifying new avenues for teacher training and the defining of guidelines for homework practices).

This study is particularly important in mathematics for the following reasons: mathematics is among the school subjects where teachers assign the largest amount of homework (e.g., Rønning, 2011; Xu, 2015), while students continue to yield worrying school results in the subject, especially in middle and high school (Gottfried et al., 2007; OECD, 2014b). Moreover, a recent meta-analysis focused on mathematics and science homework showed that the relationship between homework and academic achievement in middle school is weaker than in elementary school (Fan et al., 2017). Thus, we collected data through focus group discussions with elementary and middle school mathematics teachers in order to analyze any potential variations in their perspectives on the characteristics of quality homework, and on the characteristics of homework tasks they typically assign. Regarding the latter topic, we also collected photos of homework tasks assigned by 25% of the participating teachers in order to triangulate data and enhance the trustworthiness of our findings.

Our exploratory study was guided by the following research questions:

(1) How do elementary and middle school mathematics teachers perceive quality homework?
(2) How do elementary and middle school mathematics teachers describe the homework tasks they typically assign to students?

## Materials and Methods

### The Study Context

Despite recommendations of the need for clear homework policies (e.g., Cooper et al., 2006; Bembenutty, 2011b), Portugal has no formal guidelines for homework (e.g., concerning the frequency, length, type of tasks). Still, many teachers usually include homework as part of students’ overall grade and ask parents to monitor their children’s homework completion. Moreover, according to participants there is no specific training on homework practices for pre-service or in-service teachers.

The Portuguese educational system is organized as follows: the last two years of elementary school encompass 5th and 6th grade (10 and 11 years old), while middle school encompasses 7th, 8th, and 9th grade (12 to 14 years old). At the two school levels mentioned, mathematics is a compulsory subject and students attend three to five mathematics lessons per week depending on the duration of each class (270 min per week for Grades 5 and 6, and 225 min per week for Grades 7–9). All students are assessed by their mathematics teacher (through continuous assessment tests), and at the end of elementary and middle school levels (6th and 9th grade) students are assessed externally through a national exam that counts for 30% of the overall grade. In Portuguese schools assigning homework is a frequently used educational practice, mostly in mathematics, and usually counts toward the overall grade, ranging between 2% and 5% depending on school boards (Rosário et al., 2018).

### Participants

In the current study, all participants were involved in focus groups and 25% of them, randomly selected, were asked to submit photos of homework tasks assigned.

According to Morgan (1997), to maximize the discussion among participants it is important that they share some characteristics and experiences related to the aims of the study in question. In the current study, teachers were eligible to participate when the following criteria were met: (i) they had been teaching mathematics at elementary or middle school levels for at least two years; and (ii) they would assign homework regularly, at least twice a week, in order to have enough experiences to share in the focus group.

All mathematics teachers (*N* = 130) from 25 elementary and middle schools in Northern Portugal were contacted by email. The email informed teachers of the purposes and procedures of the study (e.g., inclusion criteria, duration of the session, session videotaping, selection of teachers to send photos of homework tasks assigned), and invited them to participate in the study. To facilitate recruitment, researchers scheduled focus group discussions considering participants’ availability. Of the volunteer teachers, all participants met the inclusion criteria. The research team did not allocate teachers with hierarchical relationships in the same group, as this might limit freedom of responses, affect the dynamics of the discussion, and, consequently, the outcomes (Kitzinger, 1995).

Initially we conducted four focus groups with elementary school teachers (5th and 6th grade, 10 and 11 years old) and four focus groups with middle school teachers (7th, 8th, and 9th grade, 12, 13 and 14 years old). Subsequently, two additional focus group discussions (one for each school level) were conducted to ensure the saturation of data. Finally, seventy-eight mathematics teachers (61 females and 17 males; an acceptance rate of 60%) from 16 schools participated in our study (see Table 1). The teachers enrolled in 10 focus groups comprised of seven to nine teachers per group. Twenty teachers were randomly selected and asked to participate in the second data collection; all answered positively to our invitation (15 females and 5 males).

**Table 1:** Participants’ demographic information.

School level	Gender	Teaching experience	Education	Workload per week
Elementary school	8 M	13–38 years	34 UG	5–15 h: 22 T
(FG 2, 4, 5, 7 and 8)	30 F		4 MD	16–25 h: 16 T
Middle school	9 M	13–38 years	34 UG	5–15 h: 5 T
(FG 1, 3, 6, 9 and 10)	31 F		6 MD	16–25 h: 27 T
				26–35 h: 8 T


*FG, focus group; M, male; F, female; UG, University Graduate; MD, Master’s Degree; T, teachers.*

According to our participants, in the school context, mathematics teachers may teach one to eight classes of different grade levels. In the current research, participants were teaching one to five classes of two or three grade levels at schools in urban or near urban contexts. The participants practiced the mandatory nationwide curriculum and a continuous assessment policy.

### Data Collection

We carried out this study following the recommendations of the ethics committee of the University of Minho. All teachers gave written informed consent to participate in the research in accordance with the Declaration of Helsinki. The collaboration involved participating in one focus group discussion, and, for 25% of the participants, submitting photos by email of the homework tasks assigned.

In the current study, aiming to deepen our comprehension of the research questions, focus group interviews were conducted to capture participants’ thoughts about a particular topic (Kitzinger, 1995; Morgan, 1997). The focus groups were conducted by two members of the research team (a moderator and a field note-taker) in the first term of the school year and followed the procedure described by Krueger and Casey (2000). To prevent mishandling the discussions and to encourage teachers to participate in the sessions, the two facilitators attended a course on qualitative research offered at their home institution specifically targeting focus group methodology.

All focus group interviews were videotaped. The sessions were held in a meeting room at the University of Minho facilities, and lasted 90 to 105 min. Before starting the discussion, teachers filled in a questionnaire with sociodemographic information, and were invited to read and sign a written informed consent form. Researchers introduced themselves, and read out the information regarding the study purpose and the focus group ground rules. Participants were ensured of the confidentiality of their responses (e.g., names and researchers’ personal notes that might link participants to their schools were deleted). Then, the investigators initiated the discussion (see Table 2). At the end of each focus group discussion, participants were given the opportunity to ask questions or make further contributions.

**Table 2:** Focus group questions.

**1. Perceived Characteristics of quality homework**
1.1. If you were asked to tell someone what homework is, how would you define/describe it?
1.2. What are the characteristics of quality homework?
**2. Characteristics of Assigned Homework**
2.1. What types of homework assignments do you usually give your students?
2.2. What are the reasons that make you give those types homework assignments?
2.3. When and how do you design homework?
2.4. How often do you assign homework?


*To ensure that the questions would be clearly understood, they were presented to two teachers from the same grade levels as the participants prior to the beginning of the study. The two teachers did not participate in the focus groups.*

After the focus group discussions, we randomly selected 25% of the participating teachers (i.e., 10 teachers from each school level), each asked to submit photos of the homework tasks assigned by email over the course of three weeks (period between two mathematics assessment tests). This data collection aimed to triangulate data from focus groups regarding the characteristics of homework usually assigned. To encourage participation, the research team sent teachers a friendly reminder email every evening throughout the period of data collection. In total, we received 125 photos (51% were from middle school teachers).

### Data Analysis

Videotapes were used to assist the verbatim transcription of focus group data. Both focus group data and photos of the homework assignments were analyzed using thematic analysis (Braun and Clarke, 2006), assisted by QSR International’s NVivo 10 software (Richards, 2005). In this analysis there are no rigid guidelines on how to determine themes; to assure that the analysis is rigorous, researchers are expected to follow a consistent procedure throughout the analysis process (Braun and Clarke, 2006). For the current study, to identify themes and sub-themes, we used the extensiveness of comments criterion (number of participants who express a theme, Krueger and Casey, 2000).

Firstly, following an inductive process one member of the research team read the first eight focus group transcriptions several times, took notes on the overall ideas of the data, and made a list of possible codes for data at a semantic level (Braun and Clarke, 2006). Using a cluster analysis by word similarity procedure in Nvivo, all codes were grouped in order to identify sub-themes and themes posteriorly. All the themes and sub-themes were independently and iteratively identified and compared with the literature on homework (Peterson and Irving, 2008). Then, the themes and sub-themes were compared with the homework characteristics already reported in the literature (e.g., Cooper, 1989; Epstein and Van Voorhis, 2001; Trautwein et al., 2006b). New sub-themes emerged from participants’ discourses (i.e., “adjusted to the availability of students,” “teachers diagnose learning”), and were grouped in the themes reported in the literature. After, all themes and sub-themes were organized in a coding scheme (for an example see Table 3). Finally, the researcher coded the two other focus group discussions, no new information was added related to the research questions. Given that the generated patterns of data were not changed, the researcher concluded that thematic saturation was reached.

**Table 3:** Examples of the coding scheme.

Theme	Sub-theme	Description
Instructional homework purposes (Cooper et al., 2006)	Practice or review	Homework aims to practice, review and consolidate the material taught in class, as well to study for tests (Epstein and Van Voorhis, 2001; Cooper et al., 2006).
	Diagnose learning (student, teacher or both) (emerging sub-theme)	Homework aims to help students, teachers or both monitor learning difficulties, and therefore adjust behaviors. Students can further study some contents and teachers can review contents and/or adjust their teaching methods. Exemplar quote: “Homework helps students understand what contents they understood or not…and this also helps me. If the students tell me that they did not understand something I can clarify the contents, correct mistakes…” (P5 FG8).
	Personal development	Homework aims to promote students’ responsibility, persistence, time management, work habits, autonomy (Epstein and Van Voorhis, 2001).
	Extension	Homework aims to develop cognitive skills and requires: knowledge and skills transference to new situations such as problem solving and projects (Cooper et al., 2006; Rosário et al., 2015).
Degree of individualization (Cooper et al., 2006) or adaptivity/adaptability (Trautwein et al., 2006a)	Student/groups of students or class	Homework tailored to meet the needs of each student or groups of students or to the class as a whole (Cooper et al., 2006). Homework adjusted to students’ knowledge (the teacher assign “different homework assignments depending on how good they are”, Trautwein et al., 2006a, p. 1103).
	Adjusted to the availability of students (emerging sub-theme)	Homework is assigned considering: students’ schedule, extracurricular activities, assessment tests or exams, the need for leisure… Exemplar quote: “If I learn that students have assessment tests during the week, I choose not to assign homework or, if it is really necessary, I just assign an exercise to be solved very quickly” (P3 FG10).


An external auditor, trained on the coding scheme, revised all transcriptions, the coding scheme and the coding process in order to minimize researchers’ biases and increase the trustworthiness of the study (Lincoln and Guba, 1985). The first author and the external auditor examined the final categorization of data and reached consensus.

Two other members of the research team coded independently the photos of the homework assignments using the same coding scheme of the focus groups. To analyze data, the researchers had to define the sub-themes “short assignments” (i.e., up to three exercises) and “long assignments” (i.e., more than three exercises). In the end, the two researchers reviewed the coding process and discussed the differences found (e.g., some exercises had several sub questions, so one of the researchers coded it as “long assignments”; see the homework sample 4 of the Supplementary Material). However, the researchers reached consensus, deciding not to count the number of sub questions of each exercise individually, because these types of questions are related and do not require a significant amount of additional time.

Inter-rater reliability (Cohen’s Kappa) was calculated. The Cohen’s Kappa was 0.86 for the data analysis of the focus groups and 0.85 for data analysis of the photos of homework assignments, which is considered very good according to Landis and Koch (1977). To obtain a pattern of data considering the school levels, a matrix coding query was run for each data source (i.e., focus groups and photos of homework assignments). Using the various criteria options in NVivo 10, we crossed participants’ classifications (i.e., school level attribute) and nodes and displayed the frequencies of responses for each row–column combination (Bazeley and Jackson, 2013).

In the end of this process of data analysis, for establishing the trustworthiness of findings, 20 teachers (i.e., ten participants of each grade level) were randomly invited, and all agreed, to provide a member check of the findings (Lincoln and Guba, 1985). Member checking involved two phases. First, teachers were asked individually to read a summary of the findings and to fill in a 5-point Likert scale (1, completely disagree; 5, completely agree) with four items: “Findings reflect my perspective regarding homework quality”; “Findings reflect my perspective regarding homework practices”; “Findings reflect what was discussed in the focus group where I participated”, and “I feel that my opinion was influenced by the other teachers during the discussion” (inverted item). Secondly, teachers were gathered by school level and asked to critically analyze and discuss whether an authentic representation was made of their perspectives regarding quality homework and homework practices (Creswell, 2007).

## Results

This study explored teachers’ perspectives on the characteristics of quality homework, and on the characteristics of the homework tasks typically assigned. To report results, we used the frequency of occurrence criterion of the categories defined by Hill et al. (2005). Each theme may be classified as “General” when all participants, or all except one, mention a particular theme; “Typical” when more than half of the cases mention a theme; “Variant” when more than 3, and less than half of the cases mention a theme; and “Rare” when the frequency is between 2 and 3 cases. In the current study, only general and typical themes were reported to discuss the most salient data.

The results section was organized by each research question. Throughout the analysis of the results, quotes from participants were presented to illustrate data. For the second research question, data from the homework assignments collected as photographs were also included.

### Initial Data Screening

All participating teachers defended the importance of completing homework, arguing that homework can help students to develop their learning and to engage in school life. Furthermore, participants also agreed on the importance of delivering this message to students. Nevertheless, all teachers acknowledged that assigning homework daily present a challenge to their teaching routine because of the heavy workload faced daily (e.g., large numbers of students per class, too many classes to teach, teaching classes from different grade levels which means preparing different lessons, administrative workload).

Teachers at both school levels talked spontaneously about the nature of the tasks they usually assign, and the majority reported selecting homework tasks from a textbook. However, participants also referred to creating exercises fit to particular learning goals. Data collected from the homework assigned corroborated this information. Most of participating teachers reported that they had not received any guidance from their school board regarding homework.

### How do Elementary and Middle School Teachers Perceive Quality Homework?

**FIGURE 1:**
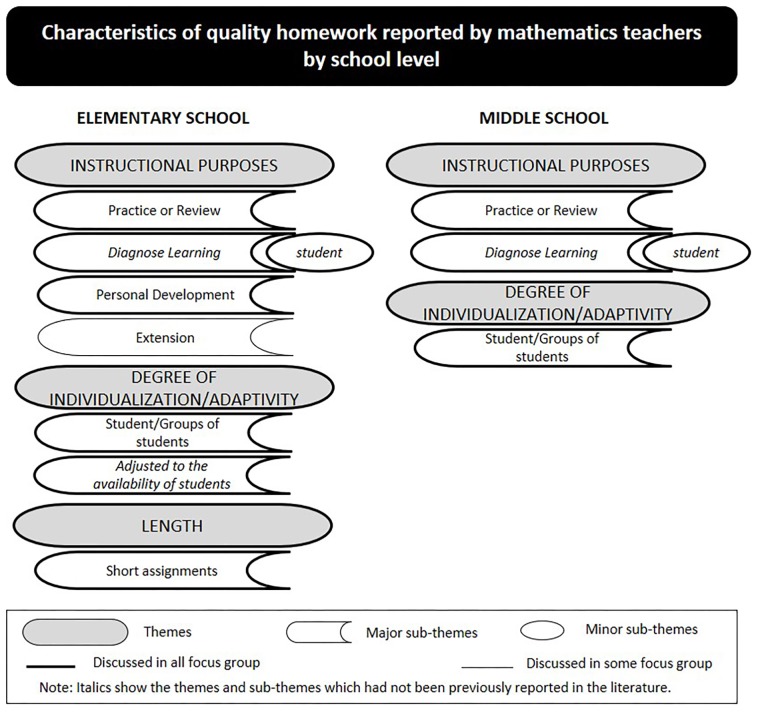
Characteristics of quality homework reported by mathematics teachers by school level.

Three main themes were identified by elementary school teachers (i.e., instructional purposes, degree of individualization/adaptivity, and length of homework) and two were identified by middle school teachers (i.e., instructional purposes, and degree of individualization/adaptivity). Figure 1 depicts the themes and sub-themes reported by teachers in the focus groups.

In all focus group discussions, all teachers from elementary and middle school mentioned “instructional purposes” as the main characteristic of quality homework. When asked to further explain the importance of this characteristic, teachers at both school levels in all focus group talked about the need for “practicing or reviewing” the content delivered in class to strengthen students’ knowledge. A teacher illustrated this idea clearly: “it is not worth teaching new content when students do not master the material previously covered” (P1 FG3). This idea was supported by participants in all focus groups; “at home they [students] have to work on the same content as those taught in class” (P1 FG7), “students have to revisit exercises and practice” (P2 FG9), “train over and over again” (P6 FG1), “practice, practice, practice” (P4 FG2).

While discussing the benefits of designing homework with the purpose of practicing the content learned, teachers at both school levels agreed on the fact that homework may be a useful tool for students to diagnose their own learning achievements while working independently. Teachers were empathetic with their peers when discussing the instrumentality of homework as a “thermometer” for students to assess their own progress. This idea was discussed in similar ways in all focus group, as the following quotation illustrates:

P2 FG1: Homework should be a bridge between class and home… students are expected to work independently, learn about their difficulties when doing homework, and check whether they understood the content.

When asked to outline other characteristics of quality homework, several elementary school teachers in all focus group mentioned that quality homework should also promote “student development” as an instructional purpose. These participants explained that homework is an instructional tool that should be designed to “foster students’ autonomy” (P9 FG4), “develop study habits and routines” (P1 FG8), and “promote organization skills and study methods” (P6 FG7). These thoughts were unanimous among participants in all focus groups. While some teachers introduced real-life examples to illustrate the ideas posited by their colleagues, others nodded their heads in agreement.

In addition, some elementary school teachers observed that homework tasks requiring transference of knowledge could help develop students’ complex thinking, a highly valued topic in the current mathematics curriculum worldwide. Teachers discussed this topic enthusiastically in two opposite directions: while some teachers defended this purpose as a characteristic of quality homework, others disagreed, as the following conversation excerpt illustrates:

P7 FG5: For me good homework would be a real challenge, like a problem-solving scenario that stimulates learning transference and develops mathematical reasoning … mathematical insight. It’s hard because it forces them [students] to think in more complex ways; still, I believe this is the type of homework with the most potential gains for them.

P3 FG5: That’s a good point, but they [students] give up easily. They just don’t do their homework. This type of homework implies competencies that the majority of students do not master…

P1 FG5: Not to mention that this type of homework takes up a lot of teaching time… explaining, checking…, and we simply don’t have time for this.

Globally, participants agreed on the potential of assigning homework with the purpose of instigating students to transfer learning to new tasks. However, participants also discussed the limitations faced daily in their teaching (e.g., number of students per class, students’ lack of prior knowledge) and concluded that homework with this purpose hinders the successful development of their lesson plans. This perspective may help explain why many participants did not perceive this purpose as a significant characteristic of quality homework. Further commenting on the characteristics of quality homework, the majority of participants at both school levels agreed that quality homework should be tailored to meet students’ learning needs. The importance of individualized homework was intensely discussed in all focus groups, and several participants suggested the need for designing homework targeted at a particular student or groups of students with common education needs. The following statements exemplifies participants’ opinions:

P3 FG3: Ideally, homework should be targeted at each student individually. For André a simple exercise, for Ana a more challenging exercise … in an ideal world homework should be tailored to students’ needs.

P6 FG6: Given the diversity of students in our classes, we may find a rainbow of levels of prior knowledge… quality homework should be as varied as our students’ needs.

As discussed in the focus groups, to foster the engagement of high-achievers in homework completion, homework tasks should be challenging enough (as reported previously by P3 FG3). However, participants at both school levels observed that their heavy daily workload prevents them from assigning individualized homework:

P1 FG1: I know it’s important to assign differentiated homework tasks, and I believe in it… but this option faces real-life barriers, such as the number of classes we have to teach, each with thirty students, tons of bureaucratic stuff we have to deal with… All this raises real-life questions, real impediments… how can we design homework tasks for individual students?

Considering this challenge, teachers from both school levels suggested that quality homework should comprise exercises with increasing levels of difficulty. This strategy would respond to the heterogeneity of students’ learning needs without assigning individualized homework tasks to each student.

While discussing individualized homework, elementary school teachers added that assignments should be designed bearing in mind students’ availability (e.g., school timetable, extracurricular activities, and exam dates). Participants noted that teachers should learn the amount of workload their students have, and should be aware about the importance of students’ well-being.

P4 FG1: If students have large amounts of homework, this could be very uncomfortable and even frustrating… They have to do homework of other subjects and add time to extracurricular activities… responding to all demands can be very stressful.

P4 FG2: I think that we have to learn about the learning context of our students, namely their limitations to complete homework in the time they have available. We all have good intentions and want them to progress, but if students do not have enough time to do their homework, this won’t work. So, quality homework would be, for example, when students have exams and the teacher gives them little or no homework at all.

The discussion about the length of homework found consensus among the elementary school teachers in all focus group in that quality homework should be “brief”. During the discussions, elementary school teachers further explained that assigning long tasks is not beneficial because “they [students] end up demotivated” (P3 FG4). Besides, “completing long homework assignments takes hours!” (P5 FG4).

### How do Elementary and Middle School Teachers Describe the Homework Tasks They Typically Assign to Students?

**FIGURE 2:**
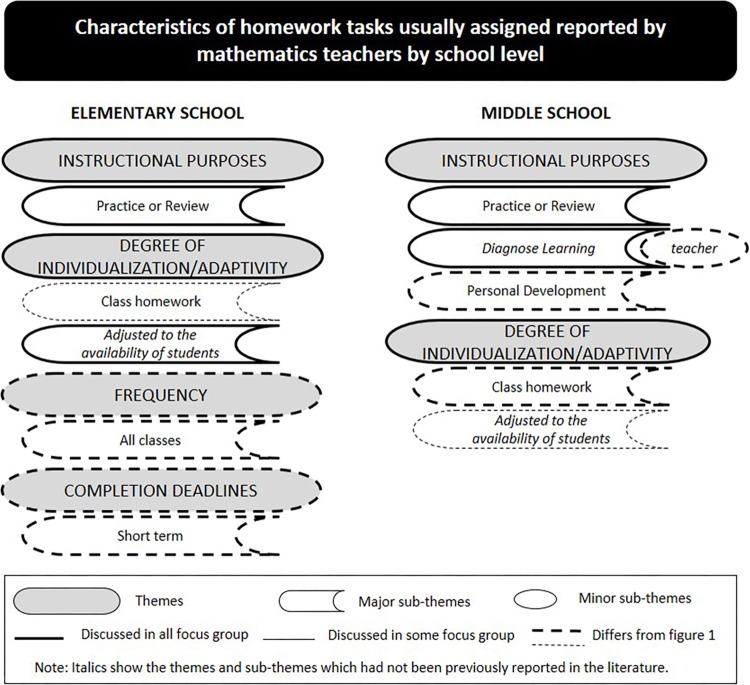
Characteristics of the homework tasks usually assigned as reported by mathematics teachers.

**FIGURE 3:**
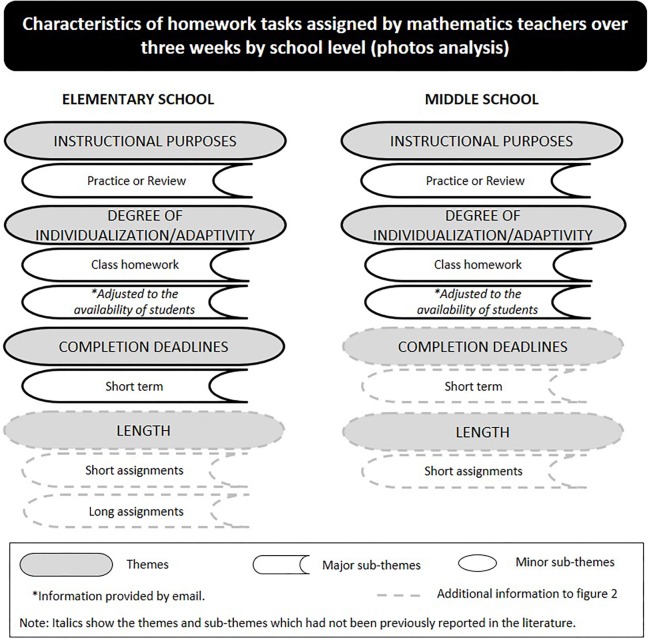
Characteristics of the homework tasks assigned by mathematics teachers.

When discussing the characteristics of the homework tasks usually assigned to their students four main themes were identified by elementary school teachers (i.e., instructional purposes, degree of individualization/adaptivity, frequency and completion deadlines), and two main themes were raised by middle school (i.e., instructional purposes, and degree of individualization/adaptivity). Figure 2 gives a general overview of the findings. Data gathered from photos added themes to findings as follows: one (i.e., length) to elementary school and two (i.e., length and completion deadlines) to middle school (see Figure 3).

While describing the characteristics of the homework tasks usually assigned, teachers frequently felt the need to compare the quality homework characteristics previously discussed with those practices. In fact, at this stage, teachers’ discourse was often focused on the analysis of the similarities and potential discrepancies found.

The majority of teachers at both school levels in all focus group reported that they assign homework with the purpose of practicing and reviewing the materials covered earlier. Participants at both school levels highlighted the need to practice the contents covered because by the end of 6th- and 9th-grade students have to sit for a national exam for which they have to be trained. This educational context may interfere with the underlying homework purposes teachers have, as this quotation illustrates:

P3 FG3: When teaching mathematics, we set several goals, but our main focus is always the final exam they [students] have to take. I like students who think for themselves, who push themselves out of their comfort zone. However, I’m aware that they have to score high on national exams, otherwise… so, I assign homework to practice the contents covered.

Beyond assigning homework with the purpose of practicing and reviewing, middle school teachers also mentioned assigning homework with the purpose of diagnosing skills and personal development (see Figure 2). Many teachers reported that they use homework as a tool to diagnose students’ skills. However, several recognized that they had previously defended the importance of homework to help students to evaluate their own learning (see Figure 1). When discussing the latter point, participants observed the need to find out about whether students had understood the content taught in class, and to decide which changes to teaching style, homework assigned, or both may be necessary.

Participant teachers at middle school in all focus groups profusely discussed the purpose of personal development when assigning homework. In fact, not many teachers at this school level mentioned this purpose as a characteristic of quality homework (it was a variant category, so it was not reported), yet it was referred to as a cornerstone in their homework practice. Reflecting on this discrepancy, middle school teachers explained in a displeased tone that their students were expected to have developed study habits and manage their school work with autonomy and responsibility. However, this “educational scenario is rare, so I feel the need to assign homework with this aim [personal development]” (P4 FG9).

Moving further in the discussion, the majority of teachers at both school levels reported to assign whole-class homework (homework designed for the whole class with no focus on special cases). “Individualized homework requires a great amount of time to be monitored” (P1 FG6), explained several participants while recalling earlier comments. Teachers justified their position referring to the impediments already mentioned (e.g., large number of students per class, number of classes from different grade levels which means preparing different lessons). Besides, teachers discussed the challenge of coping with heterogeneous classes, as one participant noted: “the class is so diverse that it is difficult to select homework tasks to address the needs of every single student. I would like to do it…but we do not live in an ideal world” (P9 FG4).

Moreover, teachers at both school levels (see Figure 2) reported to assign homework according to the availability of students; still, only elementary school teachers had earlier referred to the importance of this characteristic in quality homework. When teachers were asked to elaborate on this idea, they defended the need to negotiate with students about specific homework characteristics, for example, the amount of homework and submission deadline. In some classes, matching students’ requests, teachers might assign a “weekly homework pack” (P7 FG10). This option provides students with the opportunity to complete homework according to their availability (e.g., choosing some days during the week or weekend). Teachers agreed that ‘negotiation’ fosters students’ engagement and homework compliance (e.g., “I do not agree that students do homework on weekends, but if they show their wish and actually they complete it, for me that’s okay”, P7 FG10). In addition, teachers expressed worry about their students’ often heavy workload. Many students stay in school from 8.30 am to 6.30 pm and then attend extracurricular activities (e.g., soccer training, private music lessons). These activities leave students very little free time to enjoy as they wish, as the following statement suggests:

P8 FG4: Today I talked to a group of 5th-graders which play soccer after school three times a week. They told me that sometimes they study between 10.00 and 11.00 p.m. I was astonished. How is this possible? It’s clearly too much for these kids.

Finally, elementary school teachers in all focus group referred frequency and completion deadlines as characteristics of the homework they usually assign. The majority of teachers informed that they assign homework in almost every class (i.e., teachers reported to exclude tests eves of other subjects), to be handed in the following class.

The photos of the homework assignments (see some examples in Supplementary Material) submitted by the participating teachers served to triangulate data. The analysis showed that teachers’ discourses about the characteristics of homework assigned and the homework samples are congruent, and added information about the length of homework (elementary and middle schools) and the completion deadlines (middle school) (see Figure 3).

## Discussion and Implications for Practice and Research

Homework research have reported teachers’ perspectives on their homework practices (e.g., Brock et al., 2007; Danielson et al., 2011; Kaur, 2011; Bang, 2012; Kukliansky et al., 2014), however, literature lacks research on the quality of homework. This study adds to the literature by examining the perspectives of teachers from two school levels regarding quality homework. Moreover, participants described the characteristics of the homework assignments they typically assign, which triggered the discussion about the match between the characteristics of quality homework and the tasks actually assigned. While discussing these key aspects of the homework process, the current study provides valuable information which may help deepen our understanding of the different contributions of homework to students’ learning. Furthermore, findings are expected to inform teachers and school administrators’ homework practices and, hopefully, improve the quality of students’ learning.

All teachers at both school levels valued homework as an important educational tool for their teaching practice. Consistent with the literature, participants indicated practicing or reviewing the material covered in class as the main purpose of both the homework typically assigned (Danielson et al., 2011; Kaur, 2011) and quality homework. Despite the extended use of this homework purpose by teachers, a recent study conducted with mathematics teachers found that homework with the purpose of practicing the material covered in class did not impact significantly the academic achievement of 6th-grade students; however, homework designed with the purpose of solving problems did (extension homework) (Rosário et al., 2015). Interestingly, in the current study only teachers from elementary school mentioned the homework purpose “extension” as being part of quality homework, but these teachers did not report to use it in practice (at least it was not a typical category) (see Figure 2). Extension homework was not referenced by middle school teachers either as quality homework or as a characteristic of homework assigned. Given that middle school students are expected to master complex math skills at this level (e.g., National Research Council and Mathematics Learning Study Committee, 2001), this finding may help school administrators and teachers reflect on the value and benefits of homework to students learning progress.

Moreover, teachers at both school levels stressed the use of homework as a tool to help students evaluate their own learning as a characteristic of quality homework; however, this purpose was not said to be a characteristic of the homework usually assigned. If teachers do not explicitly emphasize this homework purpose to their students, they may not perceive its importance and lose opportunities to evaluate and improve their work.

In addition, elementary school teachers identified personal development as a characteristic of quality homework. However, only middle school teachers reported assigning homework aiming to promote students’ personal development, and evaluate students’ learning (which does not imply that students evaluate their own learning). These findings are important because existing literature has highlighted the role played by homework in promoting students’ autonomy and learning throughout schooling (Rosário et al., 2009, 2011; Ramdass and Zimmerman, 2011; Núñez et al., 2015b).

Globally, data show a disconnect between what teachers believe to be the characteristics of quality homework and the characteristics of the homework assigned, which should be further analyzed in depth. For example, teachers reported that middle school students lack the autonomy and responsibility expected for this school level, which translates to poor homework behaviors. In fact, contrary to what they would expect, middle school teachers reported the need to promote students’ personal development (i.e., responsibility and autonomy). This finding is consistent with the decrease of students’ engagement in academic activities found in middle school (e.g., Cleary and Chen, 2009; Wang and Eccles, 2012). This scenario may present a dilemma to middle school teachers regarding the purposes of homework. On one hand, students should have homework with more demanding purposes (e.g., extension); on another hand, students need to master work habits, responsibility and autonomy, otherwise homework may be counterproductive according to the participating teachers’ perspective.

Additionally, prior research has indicated that classes assigned challenging homework demonstrated high mathematics achievement (Trautwein et al., 2002; Dettmers et al., 2010). Moreover, the study by Zakharov et al. (2014) found that Russian high school students from basic and advanced tracks benefited differently from two types of homework (i.e., basic short-answer questions, and open-ended questions with high level of complexity). Results showed that a high proportion of basic or complex homework exercises enhanced mathematics exam performance for students in the basic track; whereas only a high proportion of complex homework exercises enhanced mathematics exam performance for students in the advanced track. In fact, for these students, a low proportion of complex homework exercises was detrimental to their achievement. These findings, together with our own, may help explain why the relationship between homework and mathematics achievement in middle school is lower than in elementary school (see Fan et al., 2017). Our findings suggest the need for teachers to reflect upon the importance of assigning homework to promote students’ development in elementary school, and of assigning homework with challenging purposes as students advance in schooling to foster high academic outcomes. There is evidence that even students with poor prior knowledge need assignments with some degree of difficulty to promote their achievement (see Zakharov et al., 2014). It is important to note, however, the need to support the autonomy of students (e.g., providing different the types of assignments, opportunities for students to express negative feelings toward tasks, answer students’ questions) to minimize the threat that difficult homework exercises may pose to students’ sense of competence; otherwise an excessively high degree of difficulty can lead to students’ disengagement (see Patall et al., 2018). Moreover, teachers should consider students’ interests (e.g., which contents and types of homework tasks students like) and discuss homework purposes with their students to foster their understanding of the tasks assigned and, consequently, their engagement in homework (Xu, 2010, 2018; Epstein and Van Voorhis, 2012; Rosário et al., 2018).

We also found differences between teachers’ perspectives of quality homework and their reported homework practices concerning the degree of individualization when assigning homework. Contrary to the perspectives that quality homework stresses individual needs, teachers reported to assign homework to the whole class. In spite of the educational costs associated with assigning homework adjusted to specific students or groups of students (mentioned several times by participants), research has reported benefits for students when homework assignments match their educational needs (e.g., Cooper, 2001; Trautwein et al., 2006a; Zakharov et al., 2014). The above-mentioned study by Zakharov et al. (2014) also shed light on this topic while supporting our participants’ suggestion to assign homework with increasing level of difficulty aiming to match the variety of students’ levels of knowledge (see also Dettmers et al., 2010). However, teachers did not mention this idea when discussing the characteristic of homework typically assigned. Thus, school administrators may wish to consider training teachers (e.g., using mentoring, see Núñez et al., 2013) to help them overcome some of the obstacles faced when designing and assigning homework targeting students’ individual characteristics and learning needs.

Another interesting finding is related to the sub-theme of homework adjusted to the availability of students. This was reported while discussing homework quality (elementary school) and characteristics of homework typically assigned (elementary and middle school). Moreover, some elementary and middle school teachers explained by email the reasons why they did not assign homework in some circumstances [e.g., eves of assessment tests of other subjects, extracurricular activities, short time between classes (last class of the day and next class in the following morning)]. These teachers’ behaviors show concern for students’ well-being, which may positively influence the relationship between students and teachers. As some participants mentioned, “students value this attitude” (P1 FG5). Thus, future research may explore how homework adjusted to the availability of students may contribute to encouraging positive behaviors, emotions and outcomes of students toward their homework.

Data gathered from the photos of the assigned homework tasks allowed a detailed analysis of the length and completion deadlines of homework. Long assignments did not match elementary school teachers’ perspectives of quality homework. However, a long homework was assigned once and aimed to help students practice the material covered for the mathematics assessment test. Here, practices diverged. Some teachers assigned this homework some weeks before and others assign it in last class before the test. For this reason, the “long term” completion deadline was not a typical category, hence not reported. Future research could consider studying the impact of this homework characteristic on students’ behaviors and academic performance.

Finally, our findings show that quality homework, according to teachers’ perspectives, requires attention to a combination of several characteristics of homework. Future studies may include measures to assess characteristics of homework other than “challenge” and “selection” already investigated (Trautwein et al., 2006b; Dettmers et al., 2010; Rosário et al., 2018); for example, homework adjusted to the availability of students.

### Strengths and Limitations of the Study

The current study analyzed the teachers’ perspectives on the characteristics of quality homework and of the homework they typically assigned. Despite the incapability to generalize data, we believe that these findings provide important insights into the characteristics that may impact a homework assignment’s effectiveness, especially at middle school level. For example, our results showed a disconnect between teachers’ perspectives about the characteristics of quality homework and the characteristics of the homework they assign. This finding is relevant and emphasizes the need to reflect on the consistency between educational discourses and educational practices. Teachers and school administrators could consider finding opportunities to reflect on this disconnect, which may also occur in other educational practices (e.g., teacher feedback, types of questions asked in class). Present data indicate that middle school teachers reported to assign homework with the major purpose of practicing and reviewing the material, but they also aim to develop students’ responsibility and autonomy; still they neglect homework with the purpose of extension which is focused on encouraging students to display an autonomous role, solve problems and transfer the contents learned (see discussion section). Current findings also highlight the challenges and dilemmas teachers face when they assign homework, which is important to address in teachers’ training. In fact, assigning quality homework, that is, homework that works, is not an easy task for teachers and our findings provide empirical data to discuss and reflect upon its implications for research and educational practice. Although our findings cannot be generalized, still they are expected to provide important clues to enhance teachers’ homework practices in different contexts and educational settings, given that homework is among the most universal educational practices in the classroom, is a topic of public debate (e.g., some arguments against homework are related to the characteristics of the assignments, and to the malpractices in using this educational tool) and an active area of research in many countries (Fan et al., 2017).

Moreover, these findings have identified some of the most common obstacles teachers struggle with; such data may be useful to school administrators when designing policies and to teacher training. The administrative obstacles (e.g., large number of students per class) reported by teachers may help understand some of the discrepancies found between teachers’ definition of quality homework and their actual homework practices (e.g., degree of individualization), and also identify which problems related to homework may require intervention. Furthermore, future research could further investigate this topic by interviewing teachers, videotaping classroom activities and discussing data in order to design new avenues of homework practices.

We share the perspective of Trautwein et al. (2006b) on the importance of mapping the characteristics of homework positively associated with students’ homework behaviors. Data from this study may inform future studies analyzing these relationships, promote adaptive homework behaviors and enhance learning.

Methodologically, this research followed rigorous procedures to increase the trustworthiness of findings, improving the validity of the study (e.g., Lincoln and Guba, 1985) that should be accounted for. Data from two data sources (i.e., focus groups and the homework assignments photographed) were consistent, and the member checking conducted in both phases allowed the opportunity to learn that the findings of the focus group seem to accurately reflect the overall teachers’ perspectives regarding quality homework and their homework practices.

Despite the promising contributions of this study to the body of research regarding homework practices, this specific research provides an incomplete perspective of the homework process as it has only addressed the perspectives of one of the agents involved. Future research may consider analyzing students’ perspectives about the same topic and contrast data with those of teachers. Findings are expected to help us identify the homework characteristics most highly valued by students and learn about whether they match those of teachers.

Furthermore, data from homework assignments (photos) were provided by 25% of the participating teachers and for a short period of time (i.e., three weeks in one school term). Future research may consider conducting small-scale studies by collecting data from various sources of information aiming at triangulating data (e.g., analyzing homework assignments given in class, interviewing students, conducting in-class observations) at different times of the school year. Researchers should also consider conducting similar studies in different subjects to compare data and inform teachers’ training.

Finally, our participants’ description does not include data regarding the teaching methodology followed by teachers in class. However, due to the potential interference of this variable in results, future research may consider collect and report data regarding school modality and the teaching methodology followed in class.

## Conclusion

Homework is an instructional tool that has proved to enhance students’ learning (Cooper et al., 2006; Fernández-Alonso et al., 2015; Valle et al., 2016; Fan et al., 2017; Rosário et al., 2018). Still, homework is a complex process and needs to be analyzed thoroughly. For instance, when planning and designing homework, teachers need to choose a set of homework characteristics (e.g., frequency, purposes, degree of individualization, see Cooper, 2001; Trautwein et al., 2006b) considering students’ attributes (e.g., Cooper, 2001), which may pose a daily challenge even for experienced teachers as those of the current study. Regardless of grade level, quality homework results from the balance of a set of homework characteristics, several of which were addressed by our participants. As our data suggest, teachers need time and space to reflect on their practices and design homework tasks suited for their students. To improve the quality of homework design, school administrators may consider organizing teacher training addressing theoretical models of homework assignment and related research, discussing homework characteristics and their influence on students’ homework behaviors (e.g., amount of homework completed, homework effort), and academic achievement. We believe that this training would increase teachers’ knowledge and self-efficacy beliefs to develop homework practices best suited to their students’ needs, manage work obstacles and, hopefully, assign quality homework.

## Ethics Statement

This study was reviewed and approved by the ethics committee of the University of Minho. All research participants provided written informed consent in accordance with the Declaration of Helsinki.

## Author Contributions

PR and TN substantially contributed to the conception and the design of the work. TN and JC were responsible for the literature search. JC, TN, AN, and TM were responsible for the acquisition, analysis, and interpretation of data for the work. PR was also in charge of technical guidance. JN made important intellectual contribution in manuscript revision. PR, JC, and TN wrote the manuscript with valuable inputs from the remaining authors. All authors agreed for all aspects of the work and approved the version to be published.

## Conflict of Interest Statement

The authors declare that the research was conducted in the absence of any commercial or financial relationships that could be construed as a potential conflict of interest.

## References

[B1] Bang H. (2012). Promising homework practices: teachers’ perspectives on making homework work for newcomer immigrant students. *High Sch. J.* 95 3–31. 10.1353/hsj.2012.0001

[B2] Bazeley P. Jackson K. (2013). *Qualitative Data Analysis with NVivo.* London: Sage.

[B3] Bembenutty H. (2011a). Meaningful and maladaptive homework practices: the role of self-efficacy and self-regulation. *J. Adv. Acad.* 22 448–473. 10.1177/1932202X1102200304

[B4] Bembenutty H. (2011b). The last word: an interview with Harris Cooper-Research, policies, tips, and current perspectives on homework. *J. Adv. Acad.* 22 340–350. 10.1177/1932202X1102200207

[B5] Braun V. Clarke V. (2006). Using thematic analysis in psychology. *Qual. Res. Psychol.* 3 77–101. 10.1191/1478088706qp063oa

[B6] Brock C. H. Lapp D. Flood J. Fisher D. Han K. T. (2007). Does homework matter? An investigation of teacher perceptions about homework practices for children from nondominant backgrounds. *Urban Educ.* 42 349–372. 10.1177/0042085907304277

[B7] Cleary T. J. Chen P. P. (2009). Self-regulation, motivation, and math achievement in middle school: variations across grade level and math context. *J. Sch. Psychol.* 47 291–314. 10.1016/j.jsp.2009.04.002 19712778

[B8] Cooper H. (1989). Synthesis of research on homework. *Educ. Leadersh.* 47 85–91.

[B9] Cooper H. (2001). *The Battle Over Homework: Common Ground for Administrators, Teachers, and Parents*, 2nd Edn Thousand Oaks, CA: Sage Publications.

[B10] Cooper H. Robinson J. Patall E. (2006). Does homework improve academic achievement? A synthesis of research. *Rev. Educ. Res.* 76 1–62. 10.3102/00346543076001001

[B11] Corno L. (2000). Looking at homework differently. *Element. Sch. J.* 100 529–548. 10.1086/499654

[B12] Creswell J. W. (2007). *Qualitative Inquiry and Research Method: Choosing Among Five Approaches*, 2nd Edn Thousand Oaks, CA: Sage.

[B13] Danielson M. Strom B. Kramer K. (2011). Real homework tasks: a pilot study of types, values, and resource requirements. *Educ. Res. Q.* 35 17–32.

[B14] Dettmers S. Trautwein U. Lüdtke O. (2009). The relationship between homework time and achievement is not universal: evidence from multilevel analyses in 40 countries. *Sch. Effective. Sch. Improve.* 20 375–405. 10.1080/09243450902904601

[B15] Dettmers S. Trautwein U. Lüdtke O. Kunter M. Baumert J. (2010). Homework works if homework quality is high: using multilevel modeling to predict the development of achievement in mathematics. *J. Educ. Psychol.* 102 467–482. 10.1037/a0018453

[B16] Epstein J. Van Voorhis F. (2012). “The changing debate: from assigning homework to designing homework,” in *Contemporary Debates in Child Development and Education*, eds Suggate S. Reese E. (London: Routledge), 263–273.

[B17] Epstein J. L. Van Voorhis F. L. (2001). More than ten minutes: teachers’ roles in designing homework. *Educ. Psychol.* 36 181–193. 10.1207/S15326985EP3603_4

[B18] Fan H. Xu J. Cai Z. He J. Fan X. (2017). Homework and students’ achievement in math and science: A 30-year meta-analysis, 1986–2015. *Educ. Res. Rev.* 20 35–54. 10.1016/j.edurev.2016.11.003

[B19] Fernández-Alonso R. Álvarez-Díaz M. Suárez-Álvarez J. Muñiz J. (2017). Students’ achievement and homework assignment strategies. *Front. Psychol.* 8:286. 10.3389/fpsyg.2017.00286 28326046 PMC5339273

[B20] Fernández-Alonso R. Suárez-Álvarez J. Muñiz J. (2015). Adolescents’ homework performance in mathematics and science: personal factors and teaching practices. *J. Educ. Psychol.* 107 1075–1085. 10.1037/edu0000032

[B21] Foyle H. Lyman L. Tompkins L. Perne S. Foyle D. (1990). *Homework and Cooperative Learning: A Classroom Field Experiment.* Emporia, KS: Emporia State University.

[B22] Gottfried A. E. Marcoulides G. A. Gottfried A. W. Oliver P. H. Guerin D. W. (2007). Multivariate latent change modeling of developmental decline in academic intrinsic math motivation and achievement: childhood through adolescence. *Int. J. Behav. Dev.* 31 317–327. 10.1177/0165025407077752

[B23] Hagger M. Sultan S. Hardcastle S. Chatzisarantis N. (2015). Perceived autonomy support and autonomous motivation toward mathematics activities in educational and out-of-school contexts is related to mathematics homework behavior and attainment. *Contemp. Educ. Psychol.* 41 111–123. 10.1016/j.cedpsych.2014.12.002

[B24] Hill C. E. Knox S. Thompson B. J. Williams E. N. Hess S. A. Ladany N. (2005). Consensual qualitative research: an update. *J. Couns. Psychol.* 52 196–205. 10.1037/a0033361 24295460

[B25] Hong E. Wan M. Peng Y. (2011). Discrepancies between students’ and teachers’ perceptions of homework. *J. Adv. Acad.* 22 280–308. 10.1177/1932202X1102200205

[B26] Kaur B. (2011). Mathematics homework: a study of three grade eight classrooms in Singapore. *Int. J. Sci. Math. Educ.* 9 187–206. 10.1007/s10763-010-9237-0

[B27] Kaur B. Yap S. F. Koay P. L. (2004). The learning of mathematics – expectations, homework and home support. *Primary Math.* 8 22–27.

[B28] Kitzinger J. (1995). Qualitative research: introducing focus groups. *BMJ* 311 299–302. 10.1136/bmj.311.7000.299 7633241 PMC2550365

[B29] Krueger R. A. Casey M. A. (2000). *Focus Groups: A Practical Guide for Applied Research*, 3rd Edn Thousand Oaks, CA: Sage, 10.1037/10518-189

[B30] Kukliansky I. Shosberger I. Eshach H. (2014). Science teachers’ voice on homework: beliefs, attitudes, and behaviors. *Int. J. Sci.Math. Educ.* 14 229–250. 10.1007/s10763-014-9555-8

[B31] Landis J. R. Koch G. G. (1977). The measurement of observer agreement for categorical data. *Biometrics* 33 159–174. 10.2307/2529310 843571

[B32] Lincoln Y. S. Guba E. G. (1985). *Naturalistic Inquiry.* Beverly Hills, CA: Sage.

[B33] Margolis H. McCabe P. (2004). Resolving struggling readers’ homework difficulties: a social cognitive perspective. *Read. Psychol.* 25 225–260. 10.1080/02702710490512064

[B34] Morgan D. L. (1997). *Focus Group as Qualitative Research*, 2nd Edn Thousand Oaks, CA: Sage, 10.4135/9781412984287

[B35] Muhlenbruck L. Cooper H. Nye B. Lindsay J. J. (2000). Homework and achievement: explaining the different strengths of relation at the elementary and secondary school levels. *Soc. Psych. Educ.* 3 295–317. 10.1023/A:1009680513901

[B36] National Research Council and Mathematics Learning Study Committee. (2001). *Adding it up: Helping Children Learn Mathematics.* Washington, DC: National Academies Press.

[B37] Núñez J. C. Rosário P. Vallejo G. González-Pienda J. (2013). A longitudinal assessment of the effectiveness of a school-based mentoring program in middle school. *Contemp. Educ. Psychol.* 38 11–21. 10.1016/j.cedpsych.2012.10.002

[B38] Núñez J. C. Suárez N. Cerezo R. González-Pienda J. Rosário P. Mourão R. (2015a). Homework and academic achievement across Spanish compulsory education. *Educ. Psychol.* 35 726–746. 10.1080/01443410.2013.817537

[B39] Núñez J. C. Suárez N. Rosário P. Vallejo G. Valle A. Epstein J. L. (2015b). Relationships between parental involvement in homework, student homework behaviors, and academic achievement: differences among elementary, junior high, and high school students. *Metacogn. Learn.* 10 375–406. 10.1007/s11409-015-9135-5

[B40] OECD (2014a). *PISA 2012 Results in Focus: Does Homework Perpetuate Inequities in Education?, PISA.* Paris: OECD Publishing.

[B41] OECD (2014b). *PISA 2012 Results in Focus: What 15-Year-Olds Know and what they Can do With What They Know, PISA.* Paris: OECD Publishing.

[B42] Patall E. A. Hooper S. Vasquez A. C. Pituch K. A. Steingut R. R. (2018). Science class is too hard: perceived difficulty, disengagement, and the role of teacher autonomy support from a daily diary perspective. *Learn. Instr.* 58 220–231. 10.1016/j.learninstruc.2018.07.004

[B43] Peterson E. Irving S. (2008). Secondary school students’ conceptions of assessment and feedback. *Learn. Instr.* 18 238–250. 10.1016/j.learninstruc.2007.05.001

[B44] Ramdass D. Zimmerman B. J. (2011). Developing self-regulation skills: the important role of homework. *J. Adv. Acad.* 22 194–218. 10.1177/1932202X1102200202

[B45] Richards L. (2005). *Handling Qualitative Data: A Practical Guide.* London: Sage Publications.

[B46] Rønning M. (2011). Who benefits from homework assignments? *Econ. Educ. Rev.* 30 55–64. 10.1016/j.econedurev.2010.07.001

[B47] Rosário P. Cunha J. Nunes A. R. Moreira T. Núñez C. Xu J. (2019). “Did you do your homework?” Mathematics teachers’ perspectives of homework follow-up practices at middle school. *Psychol. Sch.* 56 92–108. 10.1002/pits.22198

[B48] Rosário P. Mourão R. Baldaque M. Nunes T. Núñez J. González- Pienda J. (2009). Tareas para casa, autorregulación del aprendizaje y rendimiento en Matemáticas. *Revista de Psicodidáctica* 14 179–192.

[B49] Rosário P. Mourão R. Trigo L. Suárez N. Fernandéz E. Tuero-Herrero E. (2011). Uso de diarios de tareas para casa en el inglés como lengua extranjera: evaluación de pros y contras en el aprendizaje autorregulado y rendimiento. *Psicothema* 23 681–687. 22047858

[B50] Rosário P. Núñez J. C. Vallejo G. Cunha J. Nunes T. Mourão R. (2015). Does homework design matter? The role of homework’s purpose in student mathematics achievement. *Contemp. Educ. Psychol.* 43 10–24. 10.1016/j.cedpsych.2015.08.001

[B51] Rosário P. Núñez J. C. Vallejo G. Nunes T. Cunha J. Fuentes S. (2018). Homework purposes, homework behaviors, and academic achievement. Examining the mediating role of students’ perceived homework quality. *Contemp. Educ. Psychol.* 53 168–180. 10.1016/j.cedpsych.2018.04.001

[B52] Trautwein U. (2007). The homework-achievement relation reconsidered: differentiating homework time, homework frequency, and homework effort. *Learn. Instr.* 17 372–388. 10.1016/j.learninstruc.2007.02.009

[B53] Trautwein U. Köller O. (2003). The relationship between homework and achievement—still much of a mystery. *Educ. Psychol. Rev.* 15 115–145. 10.1023/A:1023460414243

[B54] Trautwein U. Köller O. Schmitz B. Baumert J. (2002). Do homework assignments enhance achievement? a multilevel analysis in 7th-grade mathematics. *Contemp. Educ. Psychol.* 27 26–50. 10.1006/ceps.2001.1084

[B55] Trautwein U. Lüdtke O. (2007). Students’ self-reported effort and time on homework in six school subjects: between-students differences and within-student variation. *J. Educ. Psychol.* 99 432–444. 10.1037/0022-0663.99.2.432

[B56] Trautwein U. Lüdtke O. (2009). Predicting homework motivation and homework effort in six school subjects: the role of person and family characteristics, classroom factors, and school track. *Learn. Instr.* 19 243–258. 10.1016/j.learninstruc.2008.05.001

[B57] Trautwein U. Lüdtke O. Kastens C. Köller O. (2006a). Effort on homework in grades 5 through 9: development, motivational antecedents, and the association with effort on classwork. *Child Dev.* 77 1094–1111. 10.1111/j.1467-8624.2006.00921.x 16942508

[B58] Trautwein U. Lüdtke O. Schnyder I. Niggli A. (2006b). Predicting homework effort: support for a domain-specific, multilevel homework model. *J. Educ. Psychol.* 98 438–456. 10.1037/0022-0663.98.2.438

[B59] Trautwein U. Niggli A. Schnyder I. Lüdke O. (2009a). Between-teacher differences in homework assignments and the development of students’ homework effort, homework emotions, and achievement. *J. Educ. Psychol.* 101 176–189. 10.1037/0022-0663.101.1.176

[B60] Trautwein U. Schnyder I. Niggli A. Neumann M. Lüdtke O. (2009b). Chameleon effects in homework research: the homework-achievement association depends on the measures and the level of analysis chosen. *Contemp. Educ. Psychol.* 34 77–88. 10.1016/j.cedpsych.2008.09.001

[B61] Valle A. Regueiro B. Núñez J. C. Rodríguez S. Piñeiro I. Rosário P. (2016). Academic goals, student homework engagement, and academic achievement in elementary school. *Front. Psychol.* 7:463. 10.3389/fpsyg.2016.00463 27065928 PMC4814489

[B62] Wang M.-T. Eccles J. S. (2012). Adolescent behavioral, emotional, and cognitive engagement trajectories in school and their differential relations to educational success. *J. Res. Adolesc.* 22 31–39. 10.1111/j.1532-7795.2011.00753.x

[B63] Xu J. (2010). Homework purposes reported by secondary school students: a multilevel analysis. *J. Educ. Res.* 103 171–182. 10.1080/00220670903382939

[B64] Xu J. (2015). Investigating factors that influence conventional distraction and tech-related distraction in math homework. *Comput. Educ.* 81 304–314. 10.1016/j.compedu.2014.10.024

[B65] Xu J. (2018). Reciprocal effects of homework self-concept, interest, effort, and math achievement. *Contemp. Educ. Psychol.* 55 42–52. 10.1016/j.cedpsych.2018.09.002

[B66] Xu J. Yuan R. (2003). Doing homework: listening to students’, parents’, and teachers’ voices in one urban middle school community. *Sch. Commun. J.* 13 23–44. 28407758

[B67] Zakharov A. Carnoy M. Loyalka P. (2014). Which teaching practices improve student performance on high stakes exams? Evidence from Russia. *Int. J. Educ. Dev.* 36 13–21. 10.1016/j.ijedudev.2014.01.003

