# Supplementary material for: “Homework Should Be…but We Do Not Live in an Ideal World”: Mathematics Teachers’ Perspectives on Quality Homework and on Homework Assigned in Elementary and Middle Schools
Source: Front Psychol. 2019 Feb 19;10:224. doi: 10.3389/fpsyg.2019.00224 (PMC6389699; doi:10.3389/fpsyg.2019.00224)
Supplement: Supplementary file 1 [file Table_1.docx]

Supplementary Material

“Homework should be…but we do not live in an ideal world”: Mathematics teachers’ perspectives on quality homework and on homework assigned in elementary and middle schools

Pedro Rosário*, Jennifer Cunha, Tânia Nunes, Ana Rita Nunes, Tânia Moreira, José Carlos Núñez

*** Correspondence:** Pedro Rosário: prosario@psi.uminho.pt

## Supplementary Figure


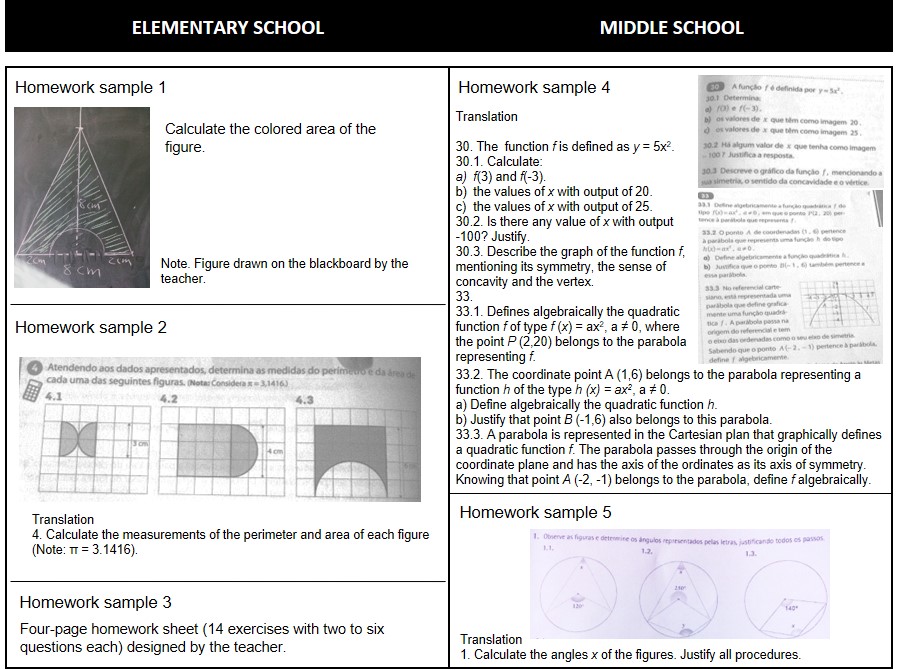


**Figure 1.** Homework samples by school level

**
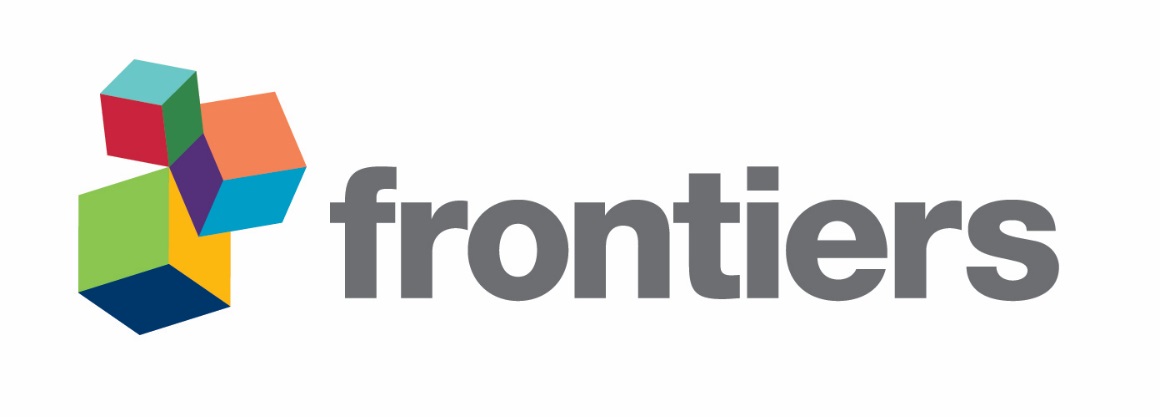
**
